# Suitability of Copper Nitride as a Wiring Ink Sintered by Low-Energy Intense Pulsed Light Irradiation

**DOI:** 10.3390/nano8080617

**Published:** 2018-08-14

**Authors:** Takashi Nakamura, Hea Jeong Cheong, Masahiko Takamura, Manabu Yoshida, Sei Uemura

**Affiliations:** 1 Research Institute for Chemical Process Technology, National Institute of Advanced Industrial Science and Technology (AIST), 4-2-1 Nigatake Miyagino-ku, Sendai, Miyagi 983-8551, Japan; 2 Flexible Electronics Research Center, National Institute of Advanced Industrial Science and Technology (AIST), 1-1-1 Higashi, Tsukuba, Ibaraki 305-8565, Japan; cheong.heajeong@imass.nagoya-u.ac.jp (H.J.C.); yoshida-manabu@aist.go.jp (M.Y.); sei-uemura@aist.go.jp (S.U.); 3 Nicca Chemical Co. Ltd., 23-1, 4-chome, Bunkyo, Fukui-city, Fukui 910-8670, Japan; m-takamura@niccachemical.com

**Keywords:** copper, copper nitride, photo sintering, ink, paste, printed electronics, post-processing

## Abstract

Copper nitride particles have a low decomposition temperature, they absorb light, and are oxidation-resistant, making them potentially useful for the development of novel wiring inks for printing circuit boards by means of intense pulsed light (IPL) sintering at low-energy. Here, we compared the thermal decomposition and light absorption of copper materials, including copper nitride (Cu_3_N), copper(I) oxide (Cu_2_O), or copper(II) oxide (CuO). Among the copper compounds examined, copper nitride had the second highest light absorbency and lowest decomposition temperature; therefore, we concluded that copper nitride was the most suitable material for producing a wiring ink that is sintered by means of IPL irradiation. Wiring inks containing copper nitride were compared with those of wiring inks containing copper nitride, copper(I) oxide, or copper(II) oxide, and copper conversion rate and sheet resistance were also determined. Under low-energy irradiation (8.3 J cm^−2^), copper nitride was converted to copper at the highest rate among the copper materials, and provided a sheet resistance of 0.506 Ω sq^−1^, indicating that copper nitride is indeed a candidate material for development as a wiring ink for low-energy intense pulsed light sintering-based printed circuit board production processes.

## 1. Introduction

Novel methods of printing circuit boards and sensing devices are currently being developed [1,2,3,4]. Compared with traditional lithographic approaches, printing circuit boards is cost-effective and allows the production of large quantities of circuit boards with less waste. In the printing of electronic circuit boards, a conductive or semiconductive wiring ink is printed onto an insulating substrate, and the ink is then subjected to heat treatment for sintering. However, thermal treatment is problematic in that it heats not only the ink but also the substrate, which can damage the final circuit board. Therefore, alternative postprocessing methods are needed.

As alternatives to heat sintering, the application of microwaves [5], infrared light [6], and laser light [7,8] have been examined. In particular, the use of intense pulsed light (IPL) irradiation has been intensely researched [9,10,11,12,13,14,15]. In IPL sintering, a short pulse of light generated by a high-power xenon flash is applied to the ink and substrate; the light generated by the xenon lamp is absorbed by the ink and converted to thermal energy, which spreads throughout the circuit by thermal diffusion and causes the ink to undergo a chemical reaction. The total time required for IPL sintering is less than 1 min; therefore, IPL sintering allows printed circuit boards to be processed at high speed.

Kim’s group is actively researching the use of IPL irradiation for the sintering of copper nanoparticles [9], silver nanoparticles [16], mixed silver and copper particles [17], and mixed copper particles of different sizes [18]. In their work using silver nanoparticles, they examined the temperature profile of a printed circuit pattern subjected to IPL irradiation and reported that the temperature of the ink was briefly increased to over 120 °C (the temperature needed for sintering of silver nanoparticles) without damaging the polyethylene terephthalate substrate (glass transition temperature = 62 °C). One reason why the substrate was protected from thermal damage during IPL sintering was that the ink was selectively heated because the ink and the substrate were colored and transparent, respectively. The temperature of the ink only was selectively increased for only a short time. Thus, in this respect, a suitable ink for IPL sintering is one that readily absorbs visible light. 

Because of their cost-effectiveness and anti-ion-migration properties compared to silver, copper and copper-containing compounds such as copper oxide, copper salts, and organic copper complexes are useful materials for producing conductive patterns [19,20,21,22]. Although copper nanoparticles are potentially useful, they easily oxidize, and harsh thermal or reduction treatments are needed to remove the oxide layer because copper(II) oxide is chemically stable. As a novel material with which to produce a wiring ink, copper nitride (Cu_3_N) may be useful because of its oxidation resistance [23] and low decomposition temperature [24]. Copper nitride has an anti-rhenium oxide structure with lattice parameters of *a* = *b* = *c* = 0.3807 nm and α = β = γ = 90° [25], and it decomposes to copper and nitrogen at around 400 °C [26]. Importantly, copper nitride is red-purple, which means that it absorbs visible light [27].

Here, we examined the suitability of using copper nitride for producing wiring inks sintered by using IPL irradiation. Dispersions containing copper nitride were prepared, and their light absorption and thermal decomposition properties were compared with those of inks containing copper nitride (Cu_3_N), copper(I) oxide (Cu_2_O), copper(II) oxide (CuO), and copper (Cu). Furthermore, two types of ink were examined: a low-viscosity liquid ink to examine the properties conferred to the ink by the copper compound, and a high-viscosity paste ink to examine a more real-world application of our ink formulations. The copper conversion ratio and sheet resistance of the inks were also determined.

## 2. Materials and Methods 

### 2.1. Materials

#### 2.1.1. Materials for Comparison with Copper Nitride

Copper(I) oxide (powder; particle size, ≤7 μm) and copper(II) oxide (nanopowder; particle size, <50 nm, as assessed by transmission electron microscopy) were purchased from Sigma-Aldrich (Tokyo, Japan). Copper nanoparticles (particle size, <100 nm) were obtained from a copper nanoparticle paste (Daiken Chemical, Osaka, Japan) by washing with acetone and then centrifuging three times.

#### 2.1.2. Materials for Preparation of Copper Nitride Nanoparticles

Copper acetate monohydrate (special grade) and 1-nonanol (special grade) were purchased from Kanto Chemical (Tokyo, Japan). Urea (special grade) and hexane (special grade) were purchased from Wako Pure Chemical Industries (Osaka, Japan). These reagents were used without further purification.

#### 2.1.3. Materials for Preparation of Vehicles for Liquid Inks

Ethanol (special grade) and ethylene glycol (special grade) were purchase from Wako Pure Chemical Industries (Osaka, Japan) and used without further purification.

#### 2.1.4. Materials for Preparation of Vehicles for Paste Inks

2-(2-Butoxyethoxy)ethyl acetate (special grade), tetraethyleneglycol monomethyl ether (95%), ethyl cellulose 45 (approximately 49% ethoxy), tetrahydrofuran (special grade), hydrochloric acid (special grade), ethyl acetate (special grade), and magnesium sulfate anhydrous (special grade) were purchased from Wako Pure Chemical Industries (Osaka, Japan). (3-Glycidoxypropyl)trimethoxysilane (GPTMS, 99.2%) was purchased from Shin-Etsu Chemical (Tokyo, Japan). Tetrahydrofuran was used after distillation to remove the stabilizer that was present in the purchase solution; the other reagents were used without further purification.

### 2.2. Preparation of Copper Nitride Nanoparticles

Copper nitride nanoparticles were prepared in accordance with previous methods [27]. Copper acetate monohydrate (20 mmol) and urea (100 mmol) were mixed with 1-nonanol (400 mL) in a three-neck flask. The atmosphere in the flask was replaced with nitrogen. The solution was heated by microwave irradiation at 190 °C for 60 min (ramp, 30 min; hold, 30 min) by using a microwave oven (μReactorEx, Shikoku Keisoku, Kagawa, Japan) equipped with a fiber optic thermometer (Anritsu Keiki, Tokyo, Japan) to obtain a liquid suspension. After the suspension was centrifuged (30,000× *g* for 60 min) and washed with hexane three times, a red-purple powder was obtained.

### 2.3. Preparation of Liquid Inks 

A sample of one of the test compounds (0.15 mg) was mixed with ethanol (1 mL) in a microcentrifuge tube, and the mixture was milled by using a homogenization pestle for 5 min. Inks containing a reductant were prepared in the same way but with the addition of ethylene glycol (0.2 mL). Aliquots (0.2 mL) of the liquid inks were dropped onto a cover glass by using a micropipette and then dried in an oven at 60 °C for 2 h (Figure S1a).

### 2.4. Synthesis of Poly(3-glycidoxypropyl)trimethoxysilane as an Adhesion Compound

Poly(3-glycidoxypropyl)trimethoxysilane (PGPTMS) was synthesized in accordance with previous methods. [28,29] Briefly, GPTMS (24.8 g), distilled water (5.4 g), and aqueous hydrochloric acid (0.1 N, 5 mL) were mixed with tetrahydrofuran (200 mL) in a flask attached to a condenser. The solution was heated under reflux for 8 h, diluted with ethyl acetate (300 mL), and then washed with distilled water three times. The organic phase was dried over anhydrous magnesium sulfate for 2 h and then filtered. A yellow viscous liquid was obtained after evaporation of the organic solvent.

### 2.5. Preparation of Paste Inks

The vehicle for the paste inks was typically prepared as follows: In a disposable plastic cup, 2-(2-butoxyethoxy)ethyl acetate (41.85 g), diethylene glycol monobutyl ether (4.65 g), ethyl cellulose (3.5 g), and PGPTMS (0.5 g) were combined and then transferred to a planetary centrifugal mixer (ARE-310; THINKY, Tokyo, Japan) and mixed at 2000 rpm for 10 min. To prepare the paste inks, each of the copper-containing materials was mixed with vehicle in a microcentrifuge tube at a weight ratio of 1:1, and the mixture was then milled by using a homogenization pestle for 5 min.

To prepare films of the paste inks, two strips of adhesive tape (thickness, 0.058 mm) were placed across a cover glass (18 × 18 mm) at an interval of 10 mm. An aliquot (approximately 0.1 mL) of paste ink was dropped onto the cover glass in the space between the two strips of adhesive tape and spread by using a glass rod (Figure S1b). The obtained ink film was dried in an oven at 60 °C for 2 h. The thickness of the prepared ink film was the thickness of the tape.

### 2.6. Intense Pulsed Light Sintering

Induced pulsed light sintering was performed by using a high-power xenon flash lamp system (SINTERON 2010-L; Xenon, MA, USA) equipped with a type B lamp. The wavelength range of the emitted light was 240–1000 nm. The experimental conditions used for the IPL sintering are indicated where necessary in the text.

### 2.7. Characterization of Copper Compounds and Inks

Diffuse reflection spectra were obtained by using a UV-3150 spectrometer (Shimadzu, Kyoto, Japan) attached to an integrating sphere (ISR-260; Shimadzu). The Kubelka–Munk equation was used to estimate the absorption ratio of the samples from the obtained reflectance. Thermogravimetric-differential thermal analysis was carried out by using a Thermo plus Evo2 instrument (Rigaku, Tokyo, Japan); samples were heated from 25 to 500 °C at 10 °C/min under air or an inert atmosphere of nitrogen, and alumina powder was used as the reference material. The crystal phase of samples was determined by means of powder X-ray diffraction analysis by using a SmartLab instrument (Rigaku) with experimental conditions of 40 kV and 30 mA from 2θ = 10° to 90°. To measure the X-ray diffraction of films prepared from the inks, the films attached with the substrate were directly placed on a zero-background holder. The morphology of the films was examined by means of scanning electron microscopy by using a TM-1000 (Hitachi, Tokyo, Japan) or S-4800 instrument (Hitachi, Tokyo, Japan) at an electron acceleration voltage of 15 kV (TM-1000) or 3 kV (S-4800), respectively. Samples were observed without a conductive coating. The sheet resistance of the films was measured by using a Loresta-GP resistivity meter (MCP-T610; Mitsubishi Chemical Analytech, Kanagawa, Japan) equipped with a four-point probe (PSP; Mitsubishi Chemical Analytech).

### 2.8. Calculation of Copper Conversion Ratio

Copper conversion ratios were estimated as follows: First, diffraction patterns obtained by means of X-ray diffraction analysis for the sintered films were fitted by using a pseudo Voigt function for peak separation. The peak separation data were then compared with reported patterns for Cu_3_N (PDF2 no. 1-73-6209), Cu (PDF2 no. 3-65-9026), CuO (PDF2 no. 1-89-5896), and Cu_2_O (PDF2 no. 1-78-2076) contained in the International Centre for Diffraction database. Next, the integral strength of the crystal planes for each crystal phase was estimated. The series of operations described above was performed by using the PDXL integrated X-ray powder diffraction software (Rigaku). Finally, the integral strengths and the following equation were used to calculate the copper conversion ratio:

$$\;R_{Cu\;conver.}=\frac{I_{Cu}}{I_{Cu3N}+I_{Cu}+I_{CuO}+I_{Cu2O}}\;$$

where *I*_Cu3N_, *I*_Cu_, *I*_CuO_, and *I*_Cu2O_ are the integral strengths for (111) of copper nitride (2θ = 40.9°), copper (2θ = 43.3°), copper(II) oxide (2θ = 38.7°), and copper(I) oxide (2θ = 36.4°), respectively.

## 3. Results and Discussion

### 3.1. Comparison of the Light Absorption and Thermal Decomposition Properties of the Copper Compounds 

**Figure 1:**
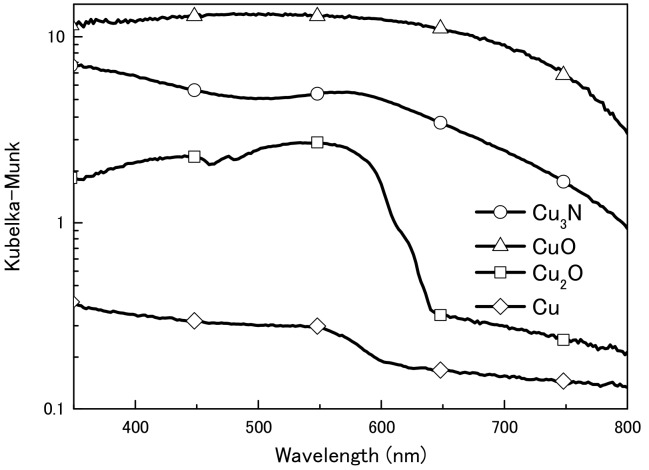
Ultraviolet–visible diffuse reflection spectra of copper nitride (Cu_3_N), copper(I) oxide (Cu_2_O), copper(II) oxide (CuO), and copper (Cu) particles.

**Figure 2:**
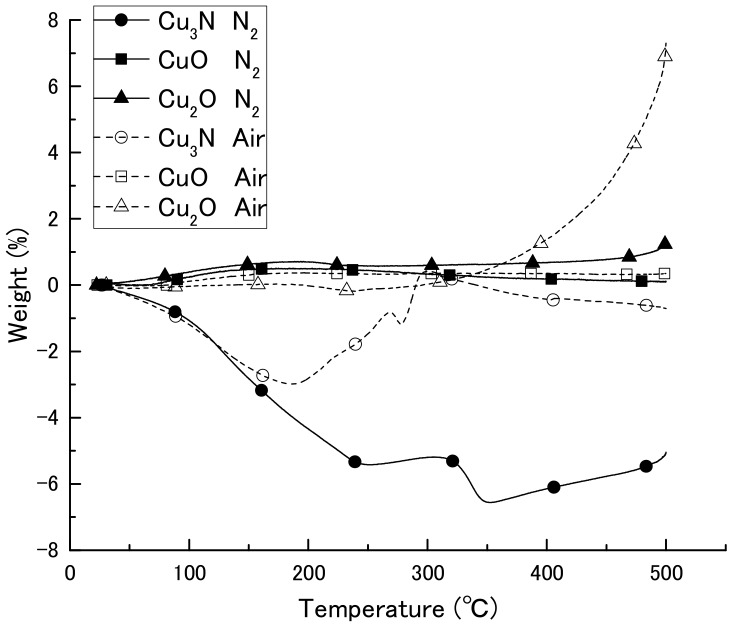
Thermogravimetric-differential thermal analysis curves of copper nitride (Cu_3_N), copper(I) oxide (Cu_2_O), and copper(II) oxide (CuO) in an air or N_2_ atmosphere.

We first obtained diffuse reflectance spectra of the copper compounds and found that diffuse reflectance increased in the order CuO > Cu_3_N > Cu_2_O > Cu (Figure 1). We then examined the thermal decomposition of Cu_3_N, CuO, and Cu_2_O under an air or N_2_ atmosphere (Figure 2). Under the N_2_ atmosphere, the weight of Cu_3_N was decreased at 100 °C, and at 250 and 500 °C the weight losses were −5.4% and −5.1%, respectively. In contrast, the weights of CuO and Cu_2_O did not change until 500 °C, at which point the weights had increased by 0.1 and 1.1%, respectively. Under the air atmosphere, the weight of copper nitride decreased until 188 °C, after which, it increased via oxidation; at 500 °C, the weight change of copper nitride was −0.7%. The weight of Cu_2_O remained constant until 333 °C at which point it began to increase as a result of oxidation; the weight was 7.2% at 500 °C. The weight of CuO remained constant, and the change was only 0.3% at 500 °C. Thus, Cu_3_N decomposed at a lower temperature than CuO and Cu_2_O, which is consistent with previous reports that CuO and Cu_2_O melt at 1193 and 1229 °C [30]. The light absorption and thermal decomposition properties of the materials are summarized in Table 1. Among the copper compounds examined, copper nitride had the second highest light absorbency and lowest decomposition temperature; therefore, we concluded that copper nitride was the most suitable material for producing a wiring ink that is sintered by means of IPL irradiation.

### 3.2. Evaluation of Liquid Inks

Simple liquid dispersions containing the copper compounds were prepared. To increase the reduction property of the copper compounds, liquid dispersions containing ethylene glycol as a reduction compound were also prepared. Films of the inks were prepared by dropping the ink onto a cover glass, and IPL sintering was performed at 12.45 and 16.60 J cm^−2^ with pulse widths of 1500 and 2000 μs, respectively; for both irradiation conditions, the applied voltage, irradiation period, and number of pulses were 2.3 kV, 1000 ms, and 1, respectively. Table 2 shows the copper conversion ratios, as estimated from the integral strength obtained by means of X-ray diffraction (Figure S2), and sheet resistances of the films made from the liquid dispersions.

In the absence of ethylene glycol, copper nitride film irradiated at 12.45 and 16.60 J cm^−2^ had copper conversion ratios of 0.91 and 0.88, respectively. However, in the presence of ethylene glycol, copper nitride film irradiated at 12.45 and 16.60 J cm^−2^ had copper conversion ratios of 0.99 and 0.96, respectively. No conversion to copper was observed for the copper(I) oxide and copper(II) oxide films, irrespective of the absence or presence of ethylene glycol.

**Figure 3:**
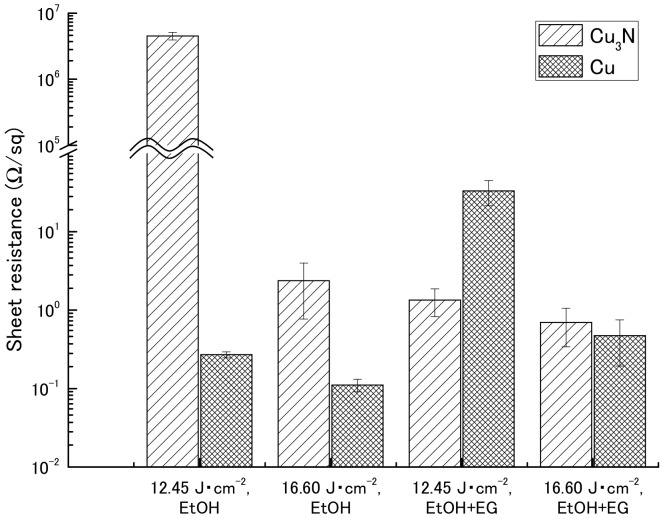
Sheet resistance of films containing copper nitride (Cu_3_N) and copper (Cu) after intense pulsed light sintering. Vehicle, ethanol (EtOH) with or without ethylene glycol (EG).

Sheet resistance after IPL sintering was measured by means of a four-point probe method in the presence or absence of ethylene glycol (Table 2 and Figure 3). The sheet resistances for the copper(I) oxide films were beyond the maximum limit of quantitation of the instrument (>9.999 × 10^7^ Ω). Similarly, sheet resistances for the copper(II) oxide films could not be obtained because the films broke apart after sintering (Figure S3).

For the copper nitride films, higher sheet resistances were obtained in the absence of ethylene glycol (4.52 × 10^6^ and 2.37 × 10^0^ Ω sq^−1^ at 12.45 and 16.60 J cm^−2^, respectively) than in the presence of ethylene glycol (1.34 × 10^0^ and 6.95 × 10^−1^ Ω sq^−1^ at 12.45 and 16.60 J cm^−2^, respectively). However, the reason for the particularly high sheet resistance obtained for the film not containing ethylene glycol and irradiated at 12.45 J cm^−2^ was that the conductive path in the film was broken by the probes during measurement, because the mechanical strength of the film was weak; visual inspection after measurement of the sheet resistance revealed that the film was broken. Compared with the sheet resistances of the films containing the copper nanoparticles, the sheet resistances of the copper nitride films were comparable or one order of magnitude higher.

**Figure 4:**
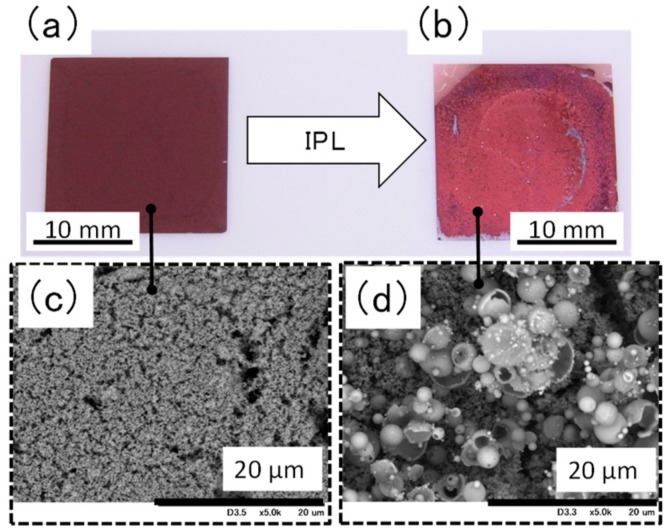
Appearance and scanning election microscopy images of a film containing ethylene glycol (**a**,**c**) before and (**b**,**d**) after intense pulsed light irradiation at 16.60 J cm^−2^.

Visual and scanning electron microscopy (Figure 4) inspection revealed that the surface of the film containing ethylene glycol became coarse after IPL irradiation at 16.60 J cm^−2^. In addition, the surface was found to contain hollow particles of copper with a particle size of 5 μm or more. The coarse particle size and failure of necking to form between particles are likely the reasons for the weakness of the film. We propose the following process for the formation of the hollow copper particles (Figure S4): Copper nitride particles are chemically changed to copper particles by the thermal energy produced by IPL irradiation; these particles rapidly fuse with nearby particles because of the decreased surface energy of the particles, and copper plate-like films form and then become round owing to surface tension. This would suggest that it is necessary to avoid rapid heating by IPL irradiation [16] and rapid cooling of the particles to prevent the generation of hollow particles. Specifically, it relaxes the heating rate to transfer the thermal energy, which is rapidly generated by IPL irradiation, to organic compound. As a result, we conclude that the condition of the film could be improved because the intense reaction is inhibited.

### 3.3. Evaluation of Paste Inks

#### 3.3.1. Use of Ethyl Cellulose and PGPTMS as a Binder and an Adhesion Reagent, respectively, to Produce a Copper Nitride Paste Ink

Although copper nitride was found to have a high copper conversion ratio, some of the films produced had a high sheet resistance because of weak mechanical strength. To address this issue, and to examine a more practical formulation for copper nitride ink, we investigated the use of a paste ink in which ethyl cellulose and poly(3-glycidoxypropyl)trimethoxysilane (PGPTMS) were used as a binder and adhesion reagent, respectively. It was previously reported that PGPTMS improves bonding strength between the particles in ink films and between the substrate and the film [28,29].

**Figure 5:**
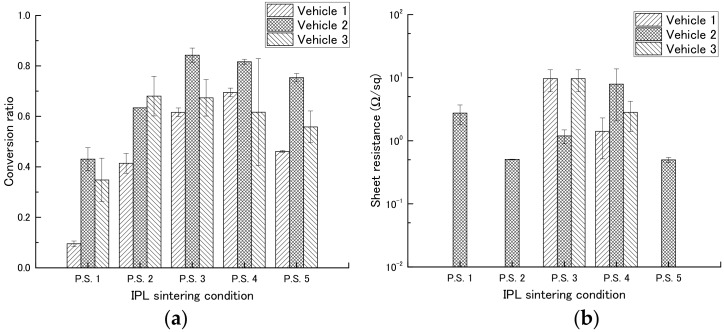
Conversion ratio (**a**) and sheet resistance (**b**) of films made with various vehicles (Table 3) and then exposed to intense pulsed light irradiation using the conditions shown in Table 4.

First, we determined the most suitable concentration of PGPTMS to use in the vehicle by preparing vehicles containing different concentrations of PGPTMS and using them to make films containing copper nitride at a weight ratio of 1:1 (Table 3). The films were then exposed to IPL irradiation under different conditions (Table 4), and copper conversion ratio and sheet resistance were measured (Table 5, Figure 5, and Figure S5).

Among the inks containing vehicles 1, 2, and 3 (PGPTMS concentrations, 0, 1, and 7 wt %, respectively), the ink containing vehicle 2 had a high copper conversion rate under most conditions. We therefore examined the effect of different IPL irradiation conditions on this ink. In a comparison of different applied voltages (2.0 [P.S.1] or 2.3 kV [P.S.2]), the copper conversion rate was increased from 0.43 to 0.63 by increasing the applied voltage. In a comparison of different pulse widths (1000 [P.S.2], 1500 [P.S.3], or 2000 μs [P.S.4]), the copper conversion rate increased with increasing pulse width, although the copper conversion rate was comparable between pulse widths of 1500 and 2000 μs. In a comparison of number of pulses (1 [P.S.2] or 4 pulses [P.S.5]), the copper conversion rate was increased from 0.63 to 0.75 by increasing the number of pulses. In addition, low sheet resistances, 5.06 × 10^−1^ and 4.97 × 10^−1^ Ω sq^−1^, were obtained for conditions P.S.2 and P.S.5, respectively. No relationship was observed between copper conversion rate and sheet resistance.

**Figure 6:**
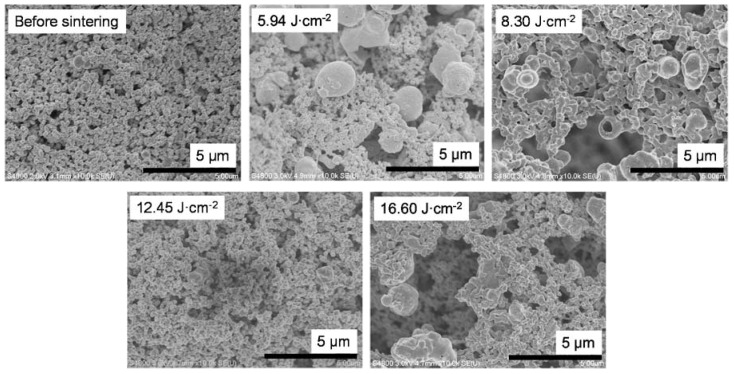
Scanning electron microscopy images of films prepared by using vehicle 2 before and after intense pulsed light sintering with different irradiation conditions.

To examine the relationship between sheet resistance and bonding between particles, the surface condition of the sintered samples prepared by using vehicle 2 was observed by means of scanning electron microscopy (Figure 6). Improved necking and no increase in particle size above micrometer size were observed in the film irradiated using the P.S.2 conditions (8.30 J cm^−2^). In addition, the influence of the additives (i.e., ethyl cellulose and PGPTMS) could be clearly observed between the paste inks (Figure 6) and the liquid inks (Figure 4). The additives inhibited the intense reaction of copper nitride by IPL irradiation (described above in the Section 3.2) because the heat energy produced by IPL irradiation spread not only to the copper nitride, but also to the additives. As a result, the formation of the particles above micrometer size with a hollow shape was inhibited. Furthermore, it appears that the necking between particles was improved by the addition of the additives because the sheet resistance was decreased and mechanical strength was increased in films containing the additives compared with those without.

In this series of experiments, the copper conversion ratio was limited to approximately 0.8 for the paste ink, owing to the penetration depth of the IPL irradiation. For all of the examined films, there was a difference in color after IPL irradiation when both sides of the film were compared (Figure S6); the back of the film was darker than the front, indicating that the copper nitride was not converted to copper at the back of the film.

#### 3.3.2. Comparison of Paste Inks Prepared with Vehicle 2

**Figure 7:**
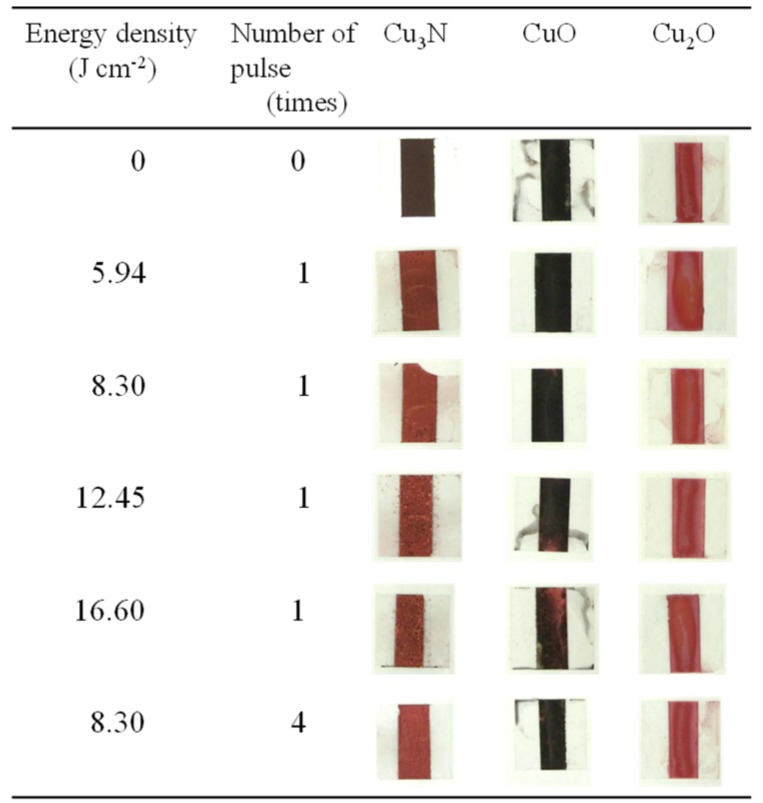
Appearance of films prepared with vehicle 2 and containing (Cu_3_N), copper(I) oxide (Cu_2_O), or copper(II) oxide (CuO) before and after intense pulsed light irradiation under the conditions shown in Table 4.

Paste inks containing copper nitride, copper(II) oxide, or copper(I) oxide were prepared with vehicle 2 and subjected to IPL sintering (sintering conditions, Table 4; copper conversion ratio and sheet resistance, Table 5; X-ray diffraction patterns, Figure S7). The appearances of the samples before and after IPL irradiation are shown in Figure 7. For the copper nitride films, the surface color was clearly changed from dark to light brown. In contrast, the color of the copper(II) oxide and copper(I) oxide films remained largely unchanged (minor changes were observed for copper(II) oxide under conditions P.S.3 and P.S.4).

The sheet resistances of the copper(II) oxide and copper(I) oxide films could not be determined because they were beyond the maximum limit of quantitation of the instrument (>9.999 × 10^7^ Ω). Thus, in the case of copper(I) oxide film, sheet resistance was not improved despite the film having improved mechanical strength compared to the film prepared by liquid ink.

### 3.4. Volume Resistivity and Comparison with that of Previous Studies

The volume resistivity of the obtained conductive film prepared by using the vehicle 2 after IPL irradiation at 8.30 J cm^−2^ was estimated. The thickness of the film after IPL irradiation was 20 μm, as determined from a cross-sectional scanning electron microscopy image (Figure S8). Assuming that 60% (Table 5) of the film from the sample surface was converted from copper nitride to copper, it is estimated that a conductive layer with a thickness of 12 μm was produced.

The results of previously published studies are shown in Table 6 together with the results of the present study. In these previously published studies, various materials (i.e., copper, copper(II) oxide, and copper organic complex) and particle shapes (i.e., nanoparticles, microparticles, and nanowire) were successfully used to produce conductive films via IPL irradiation. Although direct comparison of the results of these studies is difficult owing to differences in the spectra of the light sources used, the film in the present study had lower resistivity obtained by IPL irradiation with lower energy density than those previously reported. In the future, we intend to find the irradiation conditions and additives to obtain a conductive film with better characteristics.

## 4. Conclusions

Here, we examined the properties of films containing copper nitride, copper(II) oxide, and copper(I) oxide for their suitability as wiring inks for IPL sintering-based printed circuit board production. The following was clarified: Among the copper compounds examined, copper nitride had the second highest light absorption and lowest decomposition temperature, suggesting that it is suitable for sintering by IPL irradiation. Liquid ink containing copper nitride had a copper conversion ratio of 0.96 at an irradiation energy of 16.6 J cm^−2^. The sheet resistance of the film was on the order of 10^−1^ Ω sq^−1^, which was comparable to that of a film made from liquid ink containing copper nanoparticles. To improve the mechanical strength of the film, paste inks containing ethyl cellulose and PGPTMS as a binder and adhesion compound, respectively, were prepared. The optimum amount of PGPTMS was 1 wt %, and a film made from a paste ink containing the optimized vehicle and copper nitride and irradiated at 8.30 J cm^−2^ had a sheet resistance of 5.06 × 10^−1^ Ω sq^−1^. Together, the present results indicate that copper nitride is a suitable material for the development of wiring inks sintered by means of IPL irradiation.

## Figures and Tables

**Table 1:** Chemical properties of the copper-containing materials.

Copper-Containing Material	Light Absorbency		Decomposition Temperature or Melting Point (°C)	Copper Content Ratio
	Absorption Range (nm)	Strength		
Cu_3_N	350–800	Medium	250	93
CuO	350–800	Strong	1193	87
Cu_2_O	350–650	Medium	1229	89
Cu	350–800	Weak	1085	100

**Table 2:** Conversion ratio and sheet resistance obtained with different irradiation energy densities and vehicles.

Energy Density (J cm^−2^)	Vehicle	Conversion Ratio			Sheet Resistance (Ω sq^−1^)			
		Cu_3_N	CuO	Cu_2_O	Cu_3_N	CuO	Cu_2_O	Cu
12.45	EtOH	0.91	0	0	4.52 × 10^6^	N.D.	O.L.	2.70 × 10^−1^
16.60	EtOH	0.88	0	0	2.37 × 10^0^	N.D.	O.L.	1.11 × 10^−1^
12.45	EtOH + EG	0.99	0	0	1.34 × 10^0^	N.D.	O.L.	3.31 × 10^−1^
16.60	EtOH + EG	0.96	0	0	6.95 × 10^−1^	N.D.	O.L.	4.72 × 10^−1^

**Table 3:** Vehicle compositions.

Type of Vehicle	PGPTMS (wt %)	2-(2-Butoxyethoxy)ethyl acetate (wt %)	Diethylene Glycol Monobutyl Ether (wt %)	Ethyl Cellulose (wt %)
Vehicle 1	0	83.7	9.3	7
Vehicle 2	1	82.9	9.2	6.9
Vehicle 3	7	77.9	8.7	6.5

**Table 4:** Intense pulsed light sintering conditions.

Photo Sintering Condition	Applied Voltage (kV)	Pulse Width (μs)	Period (ms)	Number of Pulses	Total Electrical Energy (J)	Energy Density (J cm^−2^)	Distance (mm)
P.S.1	2.0	1000	1000	1	344.0	5.94	25
P.S.2	2.3	1000	1000	1	481.0	8.30	25
P.S.3	2.3	1500	1000	1	721.6	12.45	25
P.S.4	2.3	2000	1000	1	962.1	16.60	25
P.S.5	2.3	1000	1000	4	481.0	8.30	25

**Table 5:** Copper conversion ratio and sheet resistance obtained by using the intense pulsed light sintering conditions shown in Table 4.

IPL Sintering Condition	Conversion Ratio					Sheet Resistance (Ω sq^−1^)				
	Cu_3_N			CuO	Cu_2_O	Cu_3_N			CuO	Cu_2_O
Vehicle	1	2	3	2	2	1	2	3	2	2
P.S.1	0.10	0.43	0.35	0.00	0.00	O.L.	2.74 × 10^0^	O.L.	O.L.	O.L.
P.S.2	0.41	0.63	0.68	0.00	0.00	O.L.	5.06 × 10^−1^	O.L.	O.L.	O.L.
P.S.3	0.62	0.84	0.67	0.03	0.00	9.65 × 10^0^	1.19 × 10^0^	9.65 × 10^0^	O.L.	O.L.
P.S.4	0.69	0.82	0.62	0.10	0.00	1.41 × 10^0^	7.90 × 10^0^	2.82 × 10^0^	O.L.	O.L.
P.S.5	0.46	0.75	0.56	0.00	0.00	O.L.	4.97 × 10^−1^	O.L.	O.L.	O.L.

O.L. = overload.

**Table 6:** Comparison of compound information and obtained values of resistance described in the present study with previously reported values.

Entry	Compound	Form	Lower IPL Irradiation Energy			Higher IPL Irradiation Energy			References
			Energy Density (J cm^−2^)	Volume Resistivity (μΩ cm)	Sheet Resistance (mΩ sq^−1^)	Energy Density (J cm^−2^)	Volume Resistivity (μΩ cm)	Sheet Resistance (mΩ sq^−1^)	
1	Copper nitride	Nanoparticle	8.3	607	506	–	–	–	This work
2	Copper	Nanoparticle	20	Infi.	Infi.	45	5	–	[9]
3	Copper	Nanoparticle	12	Infi.	Infi.	32	173	–	[12]
4	Copper	Nanoparticle	10	–	9860	12.5	–	72	[31]
5	Copper	Nanoparticle	8.0	450	–	12.5	54	–	[32]
6	Copper	Nanoparticle + microparticle	10.0	438	–	12.5	80	–	[18]
7	Copper	Nanoparticle + nanowire	7.5	420–520	–	12.5	22.7	–	[33]
8	Copper(II) oxide	–	10.9	–	200	11	–	170	[34]
9	Copper organic complex	–	16.5	750	–	40.6	4.6	–	[11]

Infi. = infinite.

## Supplementary Materials

The following are available online at http://www.mdpi.com/2079-4991/8/8/617/s1, Figure S1: schematic images for preparing test film of (a) liquid ink and (b) paste ink, Figure S2: XRD patterns of samples for copper nitride, copper(II) oxide, and copper(I) oxide after IPL sintering, Figure S3: appearance of samples prepared from liquid ink including CuO before and after IPL sintering, Figure S4: schematic image for forming hollow particles, Figure S5: XRD patterns of different vehicles including Cu_3_N films after IPL sintering, Figure S6: appearance of a sample film after IPL sintering front side and back side, Figure S7: XRD patterns of sample films including CuO and Cu_2_O after IPL sintering, Figure S8: cross-section SEM image of the sample sintered by IPL at 8.30 J cm^−2^ of irradiation energy.
