# Supplementary material for: Suitability of Copper Nitride as a Wiring Ink Sintered by Low-Energy Intense Pulsed Light Irradiation
Source: Nanomaterials (Basel). 2018 Aug 14;8(8):617. doi: 10.3390/nano8080617 (PMC6116240; doi:10.3390/nano8080617)
Supplement: Supplementary file 1 [file nanomaterials-08-00617-s001.pdf]

Supplementary Materials

Suitability of copper nitride as a wiring ink sintered by low-energy intense pulsed light irradiation

Takashi Nakamura,[1*] Hea Jeong Cheong,[2] Masahiko Takamura,[3] Manabu Yoshida,[2] and Sei Uemura[2]

1. Research institute for chemical process technology, National Institute of Advanced Industrial Science and Technology (AIST), 4-2-1 Nigatake Miyagino-ku, Sendai, Miyagi 983-8551 Japan.
2. Flexible electronics research center, National Institute of Advanced Industrial Science and Technology (AIST), 1-1-1 Higashi, Tsukuba, Ibaraki 305-8565 Japan.
3. Nicca chemical, 23-1, 4-chome, Bunkyo, Fukui-city, Fukui 910-8670 Japan.

*Corresponding author

Email, nakamura-mw@aist.go.jp;

Tel, +81-29-861-2272;

Fax, +81-22-237-3057

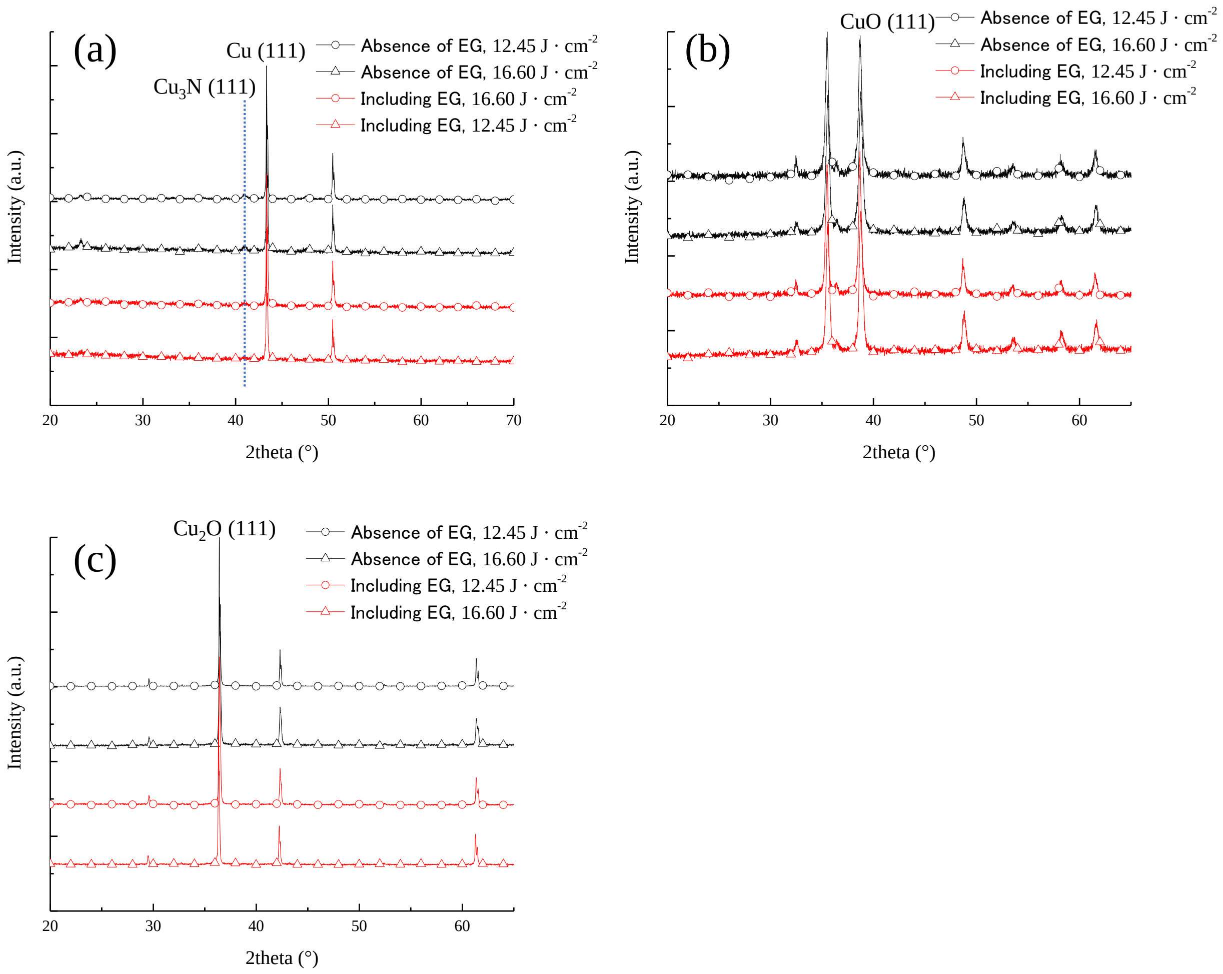

Figure S1. XRD patterns of samples for (a) copper nitride, (b) copper(II) oxide, and (c) copper(I) oxide after IPL sintering.

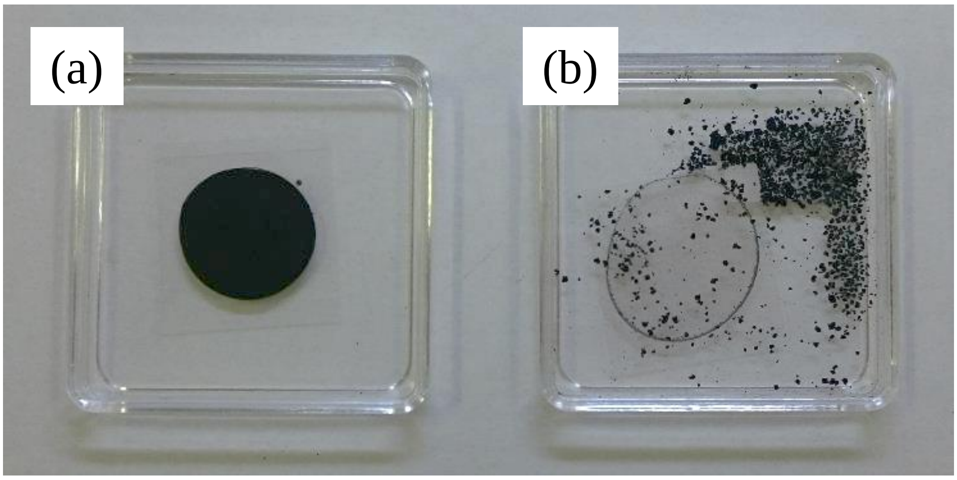

Figure S2. Appearance of samples prepared from liquid ink including CuO (a) before and (b) after IPL sintering.

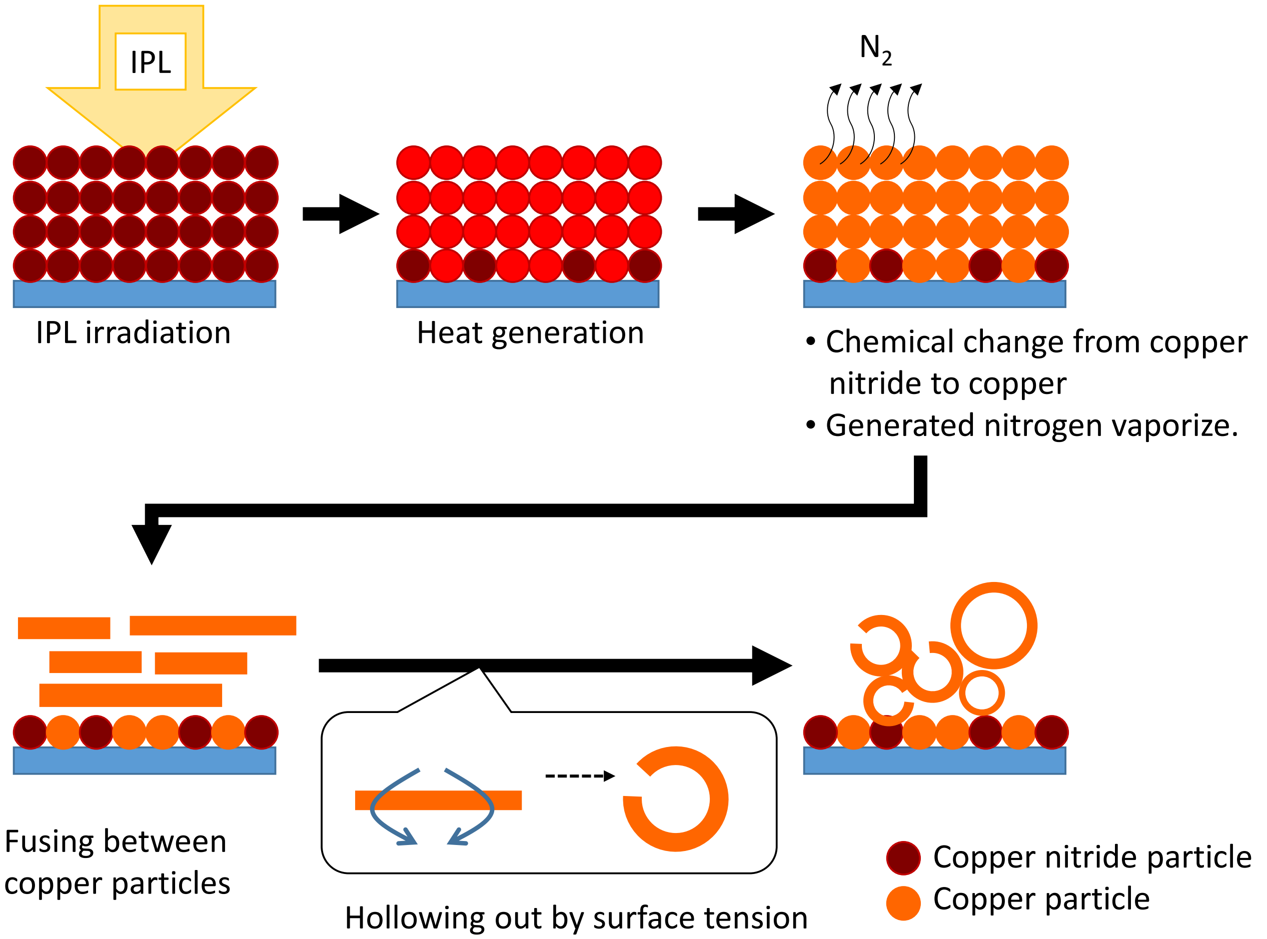

Figure S3. Schematic image for forming hollow particles.

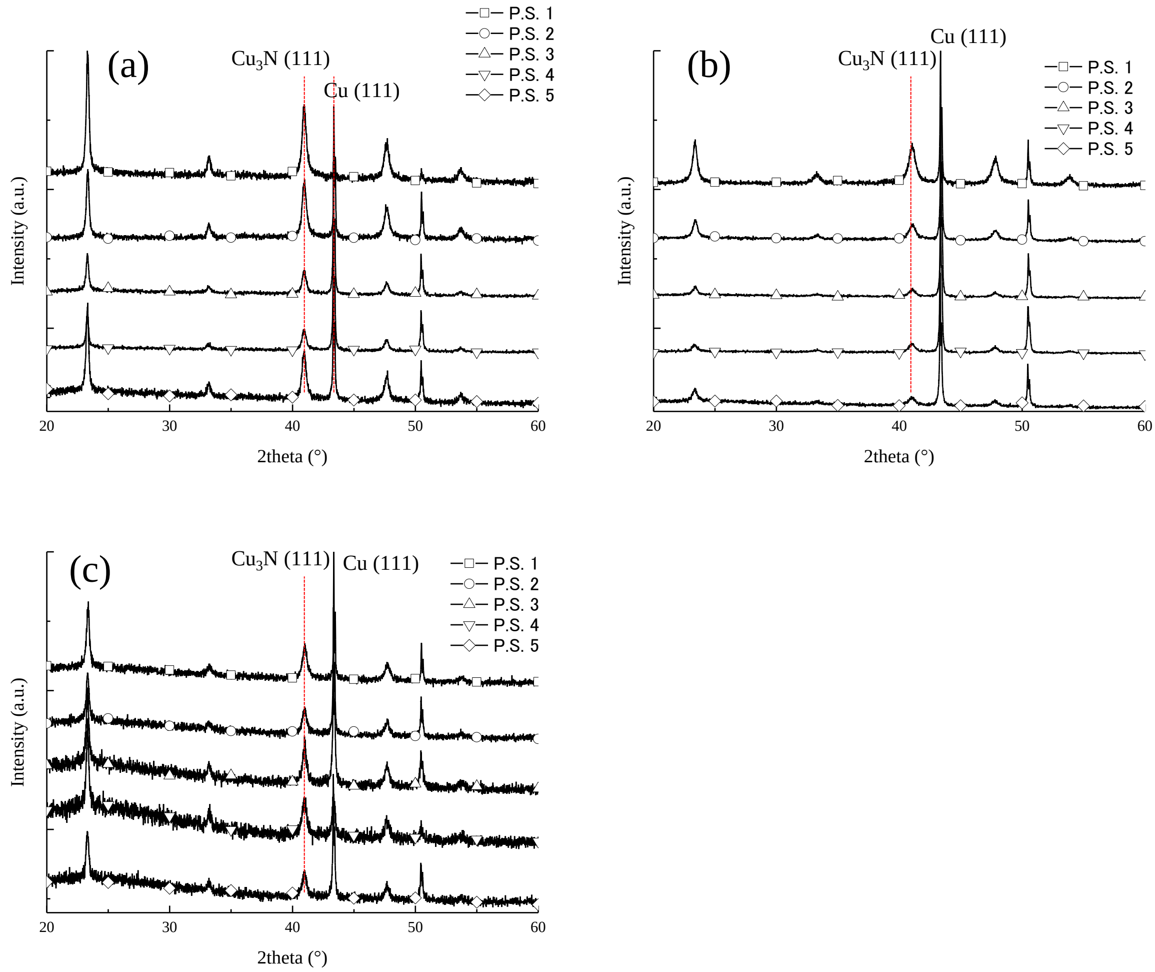

Figure S4. XRD patterns of different vehicles including Cu_3N films after IPL sintering. (a) Vehicle 1, (b) vehicle 2, and (c) vehicle 3.

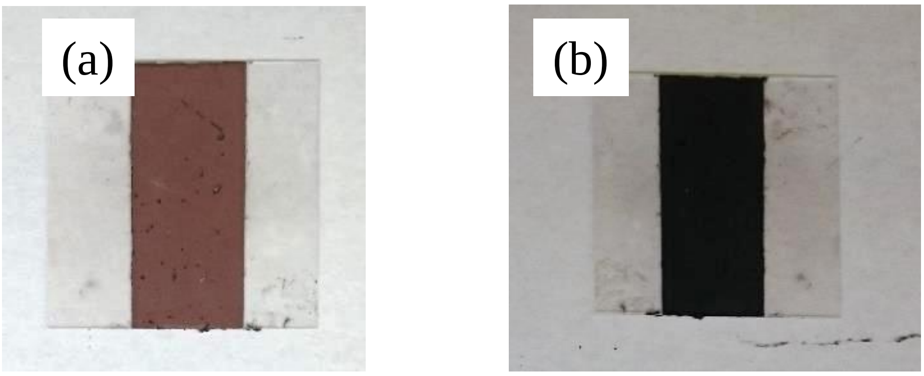

Figure S5. Appearance of a sample film after IPL sintering (a) front side and (b) back side.

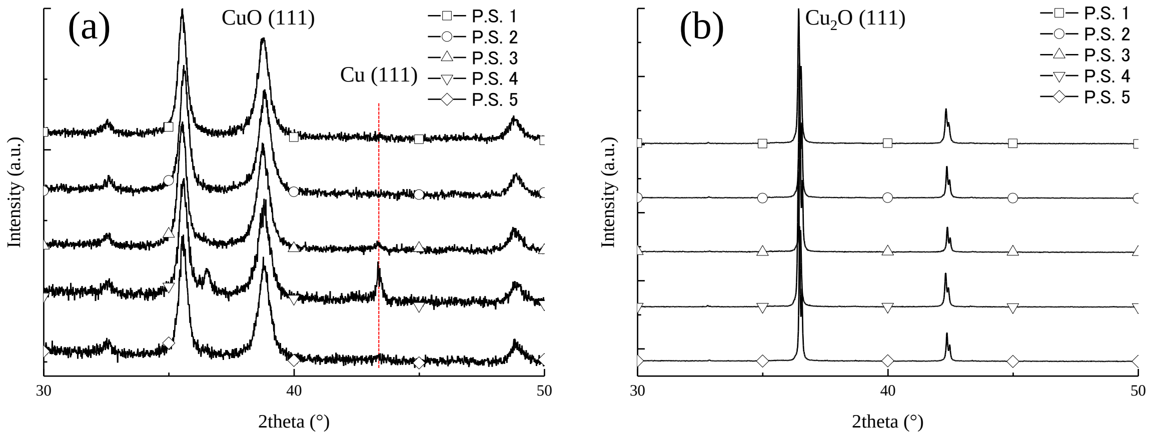

Figure S6. XRD patterns of sample films including (a) CuO and (b) Cu_2O after IPL sintering.

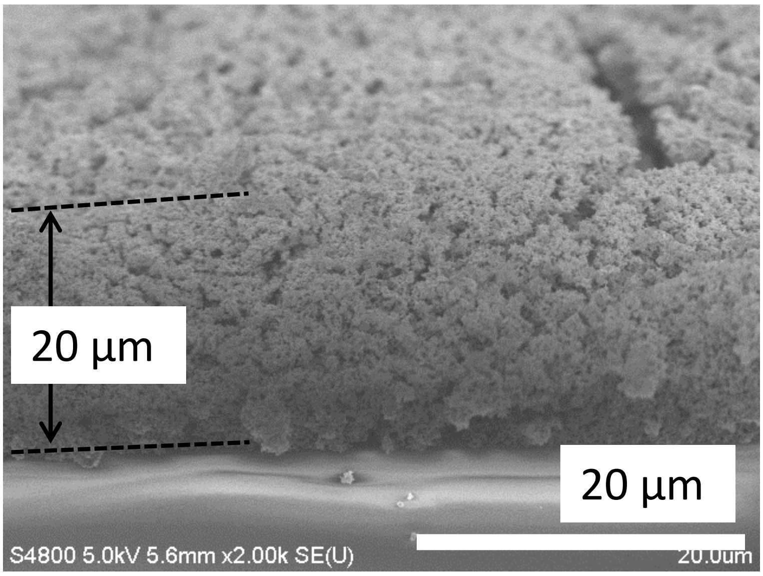

Figure S7. Cross-section SEM image of the sample sintered by IPL at 8.30 $J\cdot cm^{-2}$ of irradiation energy.
